# An Improved CenterNet Model for Insulator Defect Detection Using Aerial Imagery

**DOI:** 10.3390/s22082850

**Published:** 2022-04-08

**Authors:** Haiyang Xia, Baohua Yang, Yunlong Li, Bing Wang

**Affiliations:** 1 School of Information and Computer, Anhui Agricultural University, Hefei 230036, China; xiahaiyang@stu.ahau.edu.cn (H.X.); liyunlong@stu.ahau.edu.cn (Y.L.); 2 Anhui Provincial Key Laboratory of Power Electronics and Motion Control, Ma’anshan 243032, China; wangbing@ustc.edu; 3 School of Electrical and Information Engineering, Anhui University of Technology, Ma’anshan 243032, China

**Keywords:** insulator defect detection, CenterNet, unmanned aerial vehicle (UAV)

## Abstract

For the issue of low accuracy and poor real-time performance of insulator and defect detection by an unmanned aerial vehicle (UAV) in the process of power inspection, an insulator detection model MobileNet_CenterNet was proposed in this study. First, the lightweight network MobileNet V1 was used to replace the feature extraction network Resnet-50 of the original model, aiming to ensure the detection accuracy of the model while speeding up its detection speed. Second, a spatial and channel attention mechanism convolutional block attention module (CBAM) was introduced in CenterNet, aiming to improve the prediction accuracy of small target insulator position information. Then, three transposed convolution modules were added for upsampling, aiming to better restore the semantic information and position information of the image. Finally, the insulator dataset (ID) constructed by ourselves and the public dataset (CPLID) were used for model training and validation, aiming to improve the generalization ability of the model. The experimental results showed that compared with the CenterNet model, MobileNet_CenterNet improved the detection accuracy by 12.2%, the inference speed by 1.1 f/s for FPS-CPU and 4.9 f/s for FPS-GPU, and the model size was reduced by 37 MB. Compared with other models, our proposed model improved both detection accuracy and inference speed, indicating that the MobileNet_CenterNet model had better real-time performance and robustness.

## 1. Introduction

Insulators are extremely important components in power transmission lines. Insulators have good current insulation and mechanical protection support and undertake the function of connecting conductors and electrical insulation [1]. However, due to long-term exposure to the harsh field working environment of the natural environment, insulators are prone to defects such as cracks, breakage, and self-explosion, which lead to interruption of the power supply of the entire transmission line and seriously threaten the safe operation of the power grid [2].

The traditional power inspection method basically relies on manual labor. Skilled power workers use human eye observation and telescope observation along the transmission line to judge whether the equipment in the transmission line is faulty or normal, including insulators, anti-vibration hammers, etc. [3]. The inspection methods mentioned above not only require professional knowledge but also require sufficient physical strength and energy, which puts forward higher requirements for power security [4]. Especially for the inspection of transmission lines in mountainous areas and across large rivers, where the terrain of the lines is complex and the inspection coverage is wide. Therefore, it is difficult for manual inspection to meet the needs of power field operation and maintenance, and even power development.

The non-contact detection of power equipment has been successfully used in the detection of power equipment status, including infrared detection [5,6,7,8,9], ultraviolet detection [10], ultrasonic detection [11], infrared thermal imaging detection [12], laser detection [13], etc., which enable the state detection of power equipment to be carried out without interruption. However, data analysis and status diagnosis still rely on experienced power engineers, which limited the efficiency of power equipment status detection. Therefore, it has become a hot topic in recent years to study faster and more accurate methods for automatic detection of power equipment states.

With the development of unmanned aerial vehicles (UAVs), the method of UAV inspection has received extensive attention from many power grid companies. The identification of insulator images obtained by aerial photography is an important basis for judging the operation status of transmission lines [14]. Previous studies have shown that the use of images obtained by UAVs to extract power equipment features could effectively detect and judge defective insulators, including morphological features [15,16,17], color features [18,19], texture features [20], spatial features [21] of the insulators, etc. However, the extracted artificial features had extremely high requirements for image preprocessing, which not only consumed a lot of time but also easily led to misjudgments and missed judgments of defective insulators. Therefore, it was necessary to carry out research on the detection of insulators with automatic feature extraction.

In recent years, the development of deep learning has provided new technical ideas for intelligent inspection of power lines. Convolutional neural networks (CNN) have shown excellent capabilities in image feature representation and extraction. CNN could extract different levels of features from the input aerial image through the convolution layer, pooling layer, and fully connected layer, and achieve accurate detection of insulator targets through information classification and position regression [22]. At present, the power inspection based on the CNN model has achieved good results [23]. For example, Ling et al. successfully detected insulator states using faster R-CNN and U-net [24]. Tao et al. used ResNet-101 to achieve high-precision detection of defective insulators [25]. Two-stage detection achieved high accuracy, but still lacked in speed. Then, the one-stage model happens to solve this problem. Studies have shown that models based on one-stage have successfully detected insulators, such as SDD [26], YOLO-v2 [27], YOLO-Tiny [28], and YOLO-v3 [29]. In fact, the original one-stage detection is not a very good model either. On the one hand, the one-stage model can achieve a certain balance between detection accuracy and detection speed. On the other hand, the one-stage model generates a large number of anchor boxes on the image, especially if the image has fewer objects, which will lead to an imbalance in the number of positive and negative samples. Therefore, it is necessary to develop an optimized detection model for insulator defects.

Currently, the anchor-free-based model represented by CenterNet [30] is widely used. For example, Wu et al. used CenterNet to detect insulator defects and achieved good detection results [31]. However, the accuracy and real-time performance of insulator detection in complex background images not only required high speed and high accuracy but also required small storage capacity for easy porting to mobile devices, which would greatly promote the efficient operation of power inspection.

Therefore, in view of the sensitive issues such as the computational load and model size of the algorithm model, a lightweight insulator detection model was proposed, which combined a lightweight convolutional neural network with an anchor-free target detection network. The purpose of this research is to (1) develop a lightweight insulator detection model named MobileNet_CenterNet, aiming to reduce the parameter scale and computational complexity of the model, (2) introduce spatial and channel hybrid attention mechanism modules in different channels, utilize DIoU-NMS (distance-IoU-NMS) and add multiple transposed convolution modules, aiming to improve the detection accuracy of the model, and (3) build an insulator database, which is used to train and test the model, aiming to improve the generalizability of the model.

## 2. Materials and Methods

### 2.1. Data Processing

#### 2.1.1. Data Collection

Currently, many studies on insulator detection were based on the CPLID dataset (Chinese power line insulator dataset) provided by Tao [25], but many data from CPLID do not have the real environment. Therefore, in this study the ID (insulator dataset) is used as a self-built data set. The ID included 1983 images (5472 × 3648 pixels), which were acquired by a company in China inspecting a 500 KV overhead power line using UAVs. Among them, most of the pictures contain multiple targets, and their background information is complex, covering multiple scenes such as mountains, forests, farmlands, farms, and cities. Furthermore, due to the influence of weather conditions such as sunny, cloudy, foggy, etc., the light intensity in the picture changes greatly. In addition, during the shooting process of the drone, the insulators and their defects caused by the random shooting angle are blocked by power components such as towers. Under the influence of these factors, the detection task becomes more challenging.

**Figure 1:**
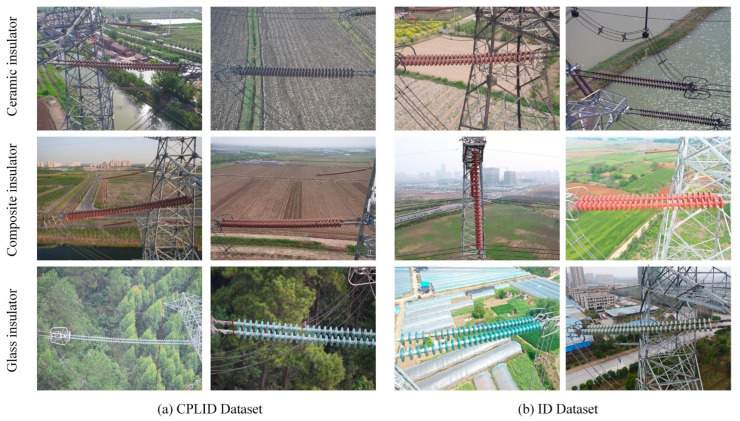
Sample images of insulators from the CPLID and ID datasets.

Figure 1 shows a partial sample of images of the CPLID and ID datasets, respectively. The first row of Figure 1 represents the ceramic insulators, the second row of Figure 1 represents the composite insulators, and the third row of Figure 1 represents the glass insulators.

#### 2.1.2. Data Labeling

**Figure 2:**
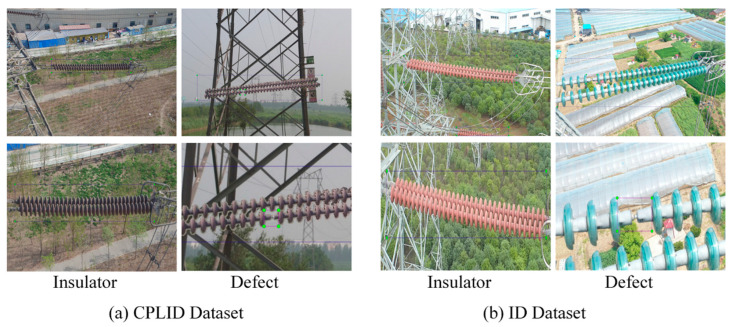
Example of insulator labeling: Insulator indicates defect-free insulator strings; Defect indicates defective insulator.

The insulator dataset constructed in this study adopted the PASCAL VOC2007 format, which consisted of a folder of images in JPG format, a folder of annotation files (xml), and a folder of image lists. The LabelImg image annotation tool (https://github.com/tzutalin/labelImg, accessed on 10 January 2022) was used to label the insulator targets with rectangular boxes, including insulator (representing defect-free insulator strings) and defect (representing defective insulator), as shown in Figure 2. Among them, the images in the first row in Figure 2 are of the original image label, and the images in the second row in Figure 2 are of the enlarged label image. Figure 2a shows an example of the labeling of the samples of the CPLID dataset, and Figure 2b shows an example of the labeling of some samples of the ID dataset.

#### 2.1.3. Training and Testing Dataset

The training set, test set, and validation set were constructed according to the ratio of 7:1:2, as shown in Table 1. For CPLID + ID dataset, the training set included 1285 images (842 for normal insulator images, 443 for defect insulator images), the validation set included 183 images (115 images for normal insulator, 68 images for defect insulator), and the test set included 363 images (265 for normal insulators, 98 for defective insulator images).

### 2.2. Basic Knowledge of CenterNet Model

CenterNet is an improved single-stage target detection model based on the CornerNet algorithm [30]. CenterNet realizes object detection by predicting the position of the center point of the object and the length and width of the corresponding object. It does not need to set anchors in advance, which greatly reduces the network parameters and the amount of calculation. CenterNet uses Resnet-50 as the backbone network to extract features and passes the extracted feature maps to the detection module, and the target is predicted by three convolution blocks, respectively, including the prediction of the center point and the category, the prediction of the target width and height, and the prediction of the center point offset.

**Figure 3:**
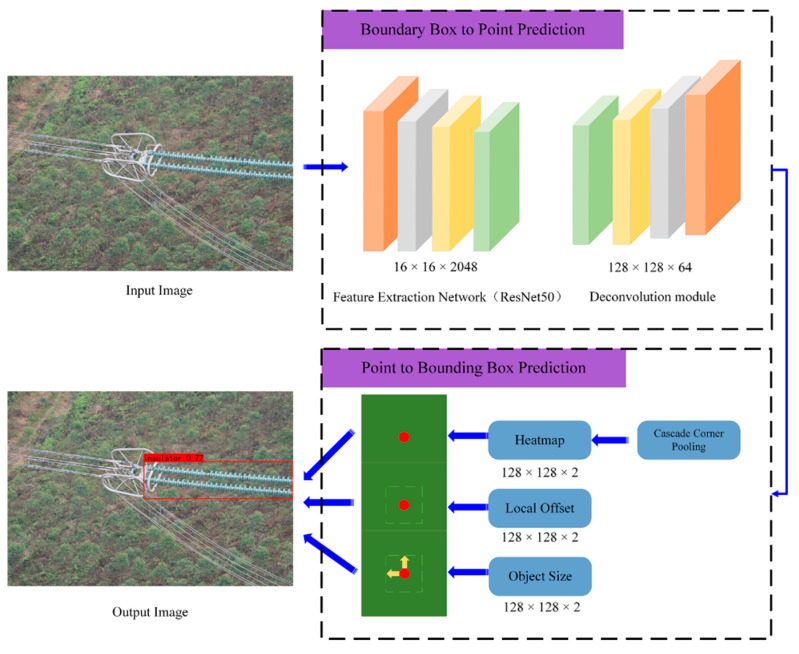
The structure of the CenterNet framework.

Therefore, the CenterNet model mainly consists of two parts, one is the prediction module from the bounding box to the point, and the other is the prediction module from the point to the bounding box, as shown in Figure 3.

The CenterNet model has the following advantages.

(1) The CenterNet model directly returned the attributes of the detection target through the detection of the position of the center point, which could realize anchor-free detection.
(2) The CenterNet model only focused on the center point information of the target, which could lead to the fast detection of the model.
(3) The CenterNet model reduced a lot of computation by extracting the local peak points of the feature map of the center point, which could make a single target have only one anchor.

### 2.3. MobileNet V1

The MobileNet V1 was proposed by Google to use depthwise separable convolutions to build lightweight deep neural networks [32], which replaced traditional convolutions with depthwise separable convolutions. The process of depthwise separable convolution is achieved by using different convolution kernels for each input channel to perform convolution, respectively, and then adjusting the channel through a 1 × 1 convolution kernel, and adding a BN (batch normalization) layer and activation function (ReLU6) after the convolution layer.

Specifically, the traditional convolution is split into a combination of depthwise convolution and pointwise convolution. In depthwise convolution, 3 convolution kernels convolve 3 channels, respectively, and obtain the features of 3 channels, respectively. Pointwise convolution is a 1 × 1 convolution of the input matrix. At the same time, compared with the traditional convolution method, the combination of depthwise convolution and pointwise convolution has greatly reduced the amount of parameters and computation. Therefore, MobileNet V1 is a lightweight convolutional neural network that can effectively maximize the recognition rate by miniaturizing parameters when computing resources are limited.

### 2.4. Convolutional Block Attention Module

The convolutional block attention module (CBAM) is a lightweight general module, which can improve the representation ability of the network without significantly increasing the network parameters [33]. It is an attention network that combines spatial and channel data, which not only considers the importance of different channels but also considers the importance of different positions of the same channel.

In this study, a dual attention mechanism is introduced to solve the difficulty in extracting the small target features of insulators. In a scene with a complex background and a large number of small target insulators, the importance of different channels and different spaces is paid attention to at the same time to improve the extraction ability of small target insulator features.

### 2.5. Insulator Detection Model Based on Mobilenet_CenterNet

#### 2.5.1. Overall Process

The insulators in the images obtained by the UAV were relatively dense, and the characteristics of the defective insulators were not significantly different. Therefore, to improve the detection accuracy of missing insulators, the MobileNet_CenterNet model was proposed. MobileNet_CenterNet used MobileNet V1 as the feature extraction network, and depthwise separable convolution as the core unit (depthwise separable convolution), including depthwise convolution and pointwise convolution, which could reduce the number of parameters of the model and the time-consuming calculation.

In addition, to obtain more effective feature maps to improve the prediction ability of missing insulator small targets and normal insulator multi-targets, MobileNet has been further improved by integrating a convolutional block attention module (CBAM), aiming to make up for the lack of contextual semantic features of targets in shallow information. To improve the detection accuracy of missing insulators, *IoU-NMS* was replaced by the *DIoU-NMS* module, which considered both the overlapping area and the center distance of the two candidate boxes in the suppression operation. The calculation formula of *DIoU-NMS* is shown in Formula (1):

(1) $$Si=\left\{ \begin{array}{ll} Si & IoU-R_{DIoU}(M,Bi)<\varepsilon \\ 0 & IoU-R_{DIoU}(M,Bi)\geq \varepsilon \\ R_{DIoU}=\frac{\rho^{2}(b,b^{gt})}{c^{2}} \end{array}\right.$$

Among them, Si is the confidence score of the current category, $R_{DIoU}$ is the penalty term of the *DIoU* loss function, Bi represents all the compared prediction boxes in the current category, M represents the box with the highest confidence in all the prediction boxes, b and $b^{gt}$ represent the coordinates of the center pixels of the two prediction boxes, c refers to the diagonal pixel length of the bounding box of the two prediction boxes, ρ represents the Euclidean distance, and ε represents the artificially set threshold, generally 0.5.

**Figure 4:**
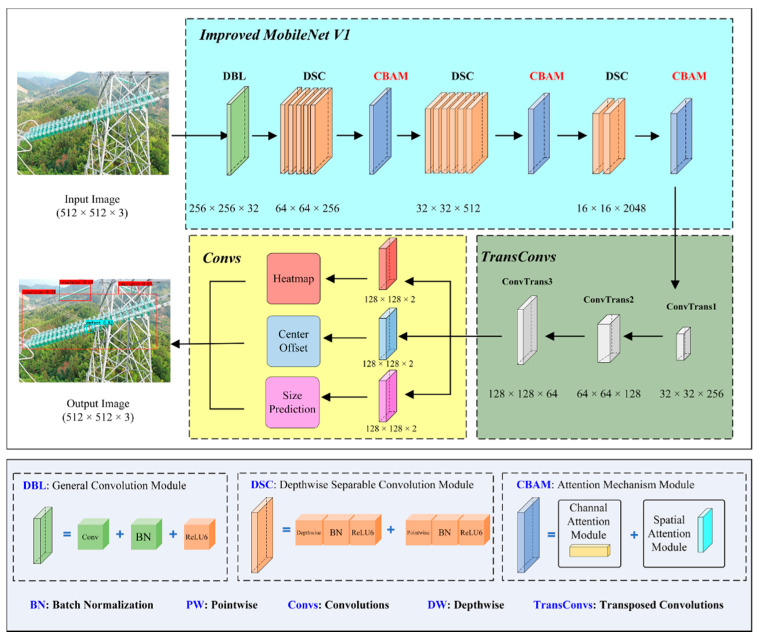
Insulator and defect detection based on MobileNet_CenterNet.

In addition, three transposed convolution (Conv2DTranspose) layers were added to achieve upsampling, aiming to better restore the semantic information and position information of the insulator image to realize the key point, bias, and size prediction of the insulator. The MobileNet_CenterNet structure is shown in Figure 4. As can be seen from Figure 4 and Table 2, the original insulator image was used as input data, and a 16 × 16 × 2048 feature map was obtained through the MobileNet V1 feature extraction network. Then, a higher resolution feature layer of 128 × 128 × 64 was obtained through the CBAM module and three transposed convolution operations. Finally, the predicted heat map, offset, width, and height were obtained, respectively. The three predictions were fused to determine the insulator bounding box, and the parameters of MobileNet_CenterNet are shown in Table 2.

#### 2.5.2. Improved Feature Extraction Network

Feature extraction network is a key part in target detection, which directly affects the detection accuracy and speed of the target detection model. There are many feature extraction networks for the original CenterNet model, such as Hourglass Net [34], DLANet [35], or ResNet [36]. These networks are mainly used to extract features from input images. Because the data set of insulator images in this study is not large enough, the size of the defect insulator objects in the images is relatively small, and there are few characteristics of insulator types. If the HourglassNet and DLANet networks with a large amount of structural parameters are used to extract features, it is easy to lead to overfitting of the model.

**Figure 5:**
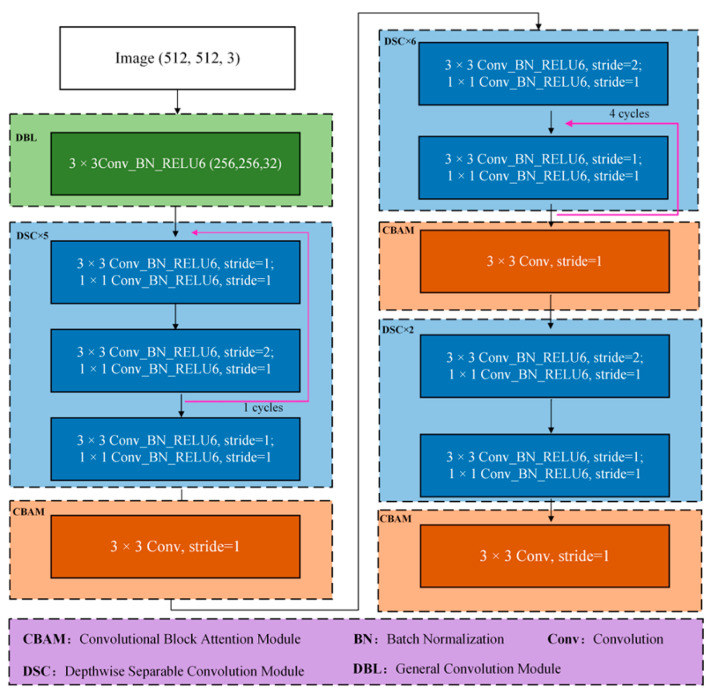
The network structure diagram of the improved MobileNetV1.

Therefore, based on the MobileNet V1 network structure in this study, a feature extraction network based on the improved CenterNet model is constructed by embedding CBAM after different depthwise separable modules. That is, the CBAM modules are introduced after the 5th, 11th, and 13th depthwise separable convolution modules, respectively. Different weights are assigned to different channels in different feature layers, which aims to improve the detection performance of the model by performing attention learning on the features of insulators and performing attention weighting operations on the features of insulator images. To improve the detection accuracy of the model, the input images were adjusted to 512 × 512 pixels using resize and padding operations as shown in Figure 5.

#### 2.5.3. Design of Loss Function

The loss function of MobileNet_CenterNet consisted of three parts, including the keypoint loss function $L_{k}$, the width and height prediction loss function $L_{off}$, and the center point offset prediction loss function $L_{size}$. The formula is shown in Equation (2):

(2) $$\;L_{sum}=L_{k}+\lambda_{off}L_{off}+\lambda_{size}L_{size}$$

Here $\lambda_{size}$ = 0.1 and $\lambda_{off}$ = 1.

The keypoint loss function $L_{k}$ was obtained by the calculation of focal loss, the formula is as shown in Formulas (3) and (4):

(3) $$L_{k}=\frac{-1}{N}\sum_{xyz}\left\{ \begin{array}{ll} {\left(1-{\bar{Y}}_{xyz}\right)}^{\alpha }log\left({\bar{Y}}_{xyz}\right) & Y_{xyz}=1\\ {\left(1-Y_{xyz}\right)}^{\beta }{\left({\bar{Y}}_{xyz}\right)}^{\alpha }log\left(1-{\bar{Y}}_{xyz}\right) & otherwise \end{array}\right.$$

(4) $$Y_{xyz}=\exp\left(-\frac{{\left(x-\bar{px}\right)}^{2}+{\left(y-\bar{py}\right)}^{2}}{2{\sigma p}^{2}}\right)\;\left(Y_{xyz}\in {\left[0,1\right]}^{\frac{w}{R}\times \frac{h}{R}\times C}\right)$$

where (x,y) represents the keypoint position of the ground-truth insulator after 4 times downsampling, z represents the category of the detected target (defect-free insulator strings and defective insulator in this study), ${\bar{Y}}_{xyz}$ represents the predicted heatmap, $Y_{xyz}$ represents the annotated ground-truth heatmap, α, β are 2 and 4, respectively, N represents the number of keypoints, $\left(\bar{px},\bar{py}\right)$ represents the coordinates of the keypoint of the insulator predicted by the thermal map, and σp represents the standard deviation.

The keypoint offset prediction loss function $L_{off}$ is shown in Formula (5):

(5) $$L_{off}=\frac{1}{N}\sum_{p}\left|\bar{O}\bar{p}-\left(\frac{p}{R}-\bar{p}\right)\right|$$

where R means zoom scale (R = 4), p represents the coordinates of the center point of the target in the image, $\bar{p}$ represents the approximate integer coordinates of the center point after scaling, $\bar{O}\bar{p}$ is the predicted position offset, and $\frac{p}{R}-\bar{p}$ is the ground-truth center point offset value.

The width and height prediction loss function $L_{size}$ is shown in Formula (6):

(6) $$L_{size}=\frac{1}{N}\sum_{k=1}^{N}\left|\bar{S}pk-S_{k}\right|$$

where pk refers to the center point of the prediction frame, $\bar{S}pk$ refers to the width and height of the prediction frame corresponding to the center point of the target k, and Sk refers to the width and height of the annotation frame corresponding to the center point of the target k.

#### 2.5.4. Determination of Heatmap Gaussian Kernel Radius

**Figure 6:**
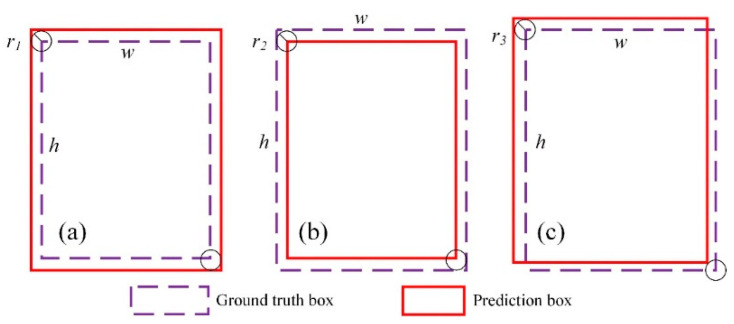
Three different position relation diagrams of the prediction box and ground truth box: (**a**) the predicted box is larger than the ground truth box, (**b**) the predicted box is smaller than the ground truth box, and (**c**) the predicted box and the ground truth box are partially superimposed.

During the training process of the original CenterNet model, there are generally three positional relationships between the prediction box and the ground truth box, as shown in Figure 6. Among them, case 1: the two corners of the prediction box and the ground truth box are circumscribed with a radius of r1. Case 2: the two corners of the prediction box and the ground truth box are inscribed in a circle with a radius of r2. Case 3: the two corners of the prediction box and the ground truth box are inscribed on one side and circumscribed on the other side of the circle with a radius of r3.

As can be seen from Figure 6, the prediction boxes in all three cases can well surround the target. To obtain a better prediction frame, the Gaussian kernel radius in Figure 6 needs to be calculated more accurately.

The calculation of the Gaussian kernel radius r is shown in Formula (6).

(7) r=min(r1,r2,r3)

For Figure 6a:

(8) $$I_{overlap}=\frac{S_{overlap}}{S_{union}}=\frac{w\times h}{\left(w+2r1)\times (h+2r1\right)}$$

(9) $$r1=\frac{-\left(w+h\right)+\sqrt{{\left(w+h\right)}^{2}+4wh\frac{I_{overlap}-1}{I_{overlap}}}}{4}$$

For Figure 6b:

(10) $$I_{overlap}=\frac{S_{overlap}}{S_{union}}=\frac{\left(w-2r2)(h-2r2\right)}{w\times h}$$

(11) $$r2=\frac{2\left(w+h\right)+\sqrt{4{\left(w+h\right)}^{2}-16\left(1-I_{overlap}\right)wh}}{8}$$

For Figure 6c:

(12) $$I_{overlap}=\frac{S_{overlap}}{S_{union}}=\frac{\left(h-r3)(w-r3\right)}{\left(h-r3)(w-r3)+2[wr3+(h-r3)r3\right]}$$

(13) $$r3=\frac{(w+h)+\sqrt{{\left(w+h\right)}^{2}-\frac{4(1-I_{overlap})}{1+I_{overlap}}}}{2}$$

Here, $S_{overlap}$ represents the overlapping part of the predicted box and the ground truth box, and $S_{union}$ represents the union of the predicted box and the ground truth box. $I_{overlap}$ represents the ratio of $S_{overlap}$ to $S_{union}$, which is taken as 0.7 in this study. w and h represent the width and height of the ground truth box.

#### 2.5.5. Model Evaluation Metrics

To effectively evaluate the insulator detection model, precision, recall, F1, and mAP (mean average precision) were used in this study [37]. In addition, the processing speed of the model was evaluated by *FPS* (frame per second):

(14) $$FPS=\frac{N}{\sum_{j=1}^{N}T_{j}}$$

Among them, N is the number of pictures and $T_{j}$ is the time required by the algorithm to process the jth image.

## 3. Results

### 3.1. Experimental Environment and Model Training

The experiments in this study were based on the MobileNet_CenterNet network built by the Pytorch deep learning framework. The operating system was Windows10, NVIDIA GeForce GTX 1070Ti GPU/8 G, Intel Core i7-8700 CPU, 16 G memory. The running program software Pycharm2019, Python 3.6 (https://www.python.org/, accessed on 10 January 2022), CUDA 10.1, and Cudnn 7.5.1 were installed for deep learning acceleration operations. The deep learning framework is Pytorch1.4, Opencv3.4, and a series of libraries such as numpy1.18.4 to assist code running.

Part of the parameter settings for model training were as follows. Epoch: 1600, Batch_size: 8, learning rate: 1 × 10^3^, Nms_threhold: 0.3, and confidence: 0.3.

To improve the robustness of the model, the MobileNet_CenterNet network was trained and iterated 1600 times using a mixed dataset composed of public data and self-built datasets, and the weights were saved every 10 times of training. The weight of the 1599th iteration was selected as the weight of the optimal model.

### 3.2. Detection Results with Different Data

To verify the detection effect of MobileNet_CenterNet, three different datasets were used for training and testing, and the detection results are shown in Table 3. For the detection result of missing insulators, the AP was 0.794–0.837, and the precision was 0.958–0.991. For normal insulator string testing, the AP was 0.966–0.979, and the precision was 0.971–0.989.

For the results of training and testing the MobileNet_CenterNet model with different datasets, the AP and precision values of defect detection for the dataset CPLID + ID with 1831 images increased by 5.1% and 3.3% compared with the dataset CPLID with 848 images, which the dataset ID of 983 images increased by 1.3% and 1.8%.

The values of AP and precision for insulator string detection for the dataset CPLID + ID with 1831 images increased by 2.3% and 1.8% over the dataset CPLID with 848 images, and increased by 0.7% and 0.7% over the dataset ID with 983 images. The results showed that the more images included in the dataset, the more accurate the model is trained. Figure 8 showed the test results of insulators of different materials.

### 3.3. Detection Results in Different Challenges Scenarios

**Figure 7:**
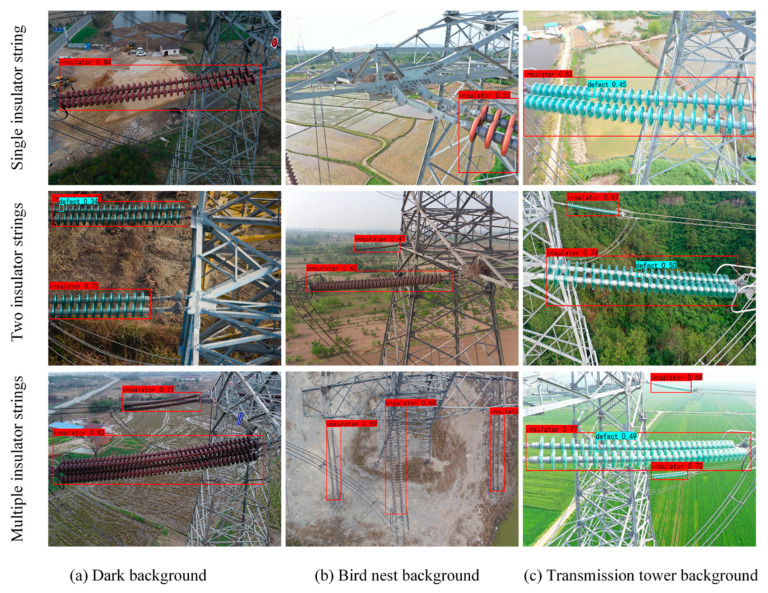
Comparison of detection results of insulators in different background environments.

To further verify the effectiveness of the MobileNet_CenterNet model proposed in this paper, insulator pictures of different background environments were taken for testing, and the results are shown in Figure 7. It can be seen from Figure 7 that the model proposed in this study can accurately detect normal insulator strings and missing insulators regardless of whether the background light was bright or dark, or there were iron towers and bird nests in the background.

In addition, better detection results were obtained through the detection of different numbers of insulator strings, including one insulator string, two insulator strings, and multiple insulator strings. Experiments showed that the MobileNet_CenterNet model proposed in this study had good robustness and could adapt to insulator detection in various background environments.

## 4. Discussion

### 4.1. Comparison of Detection Effects before and after Model Optimization

**Figure 8:**
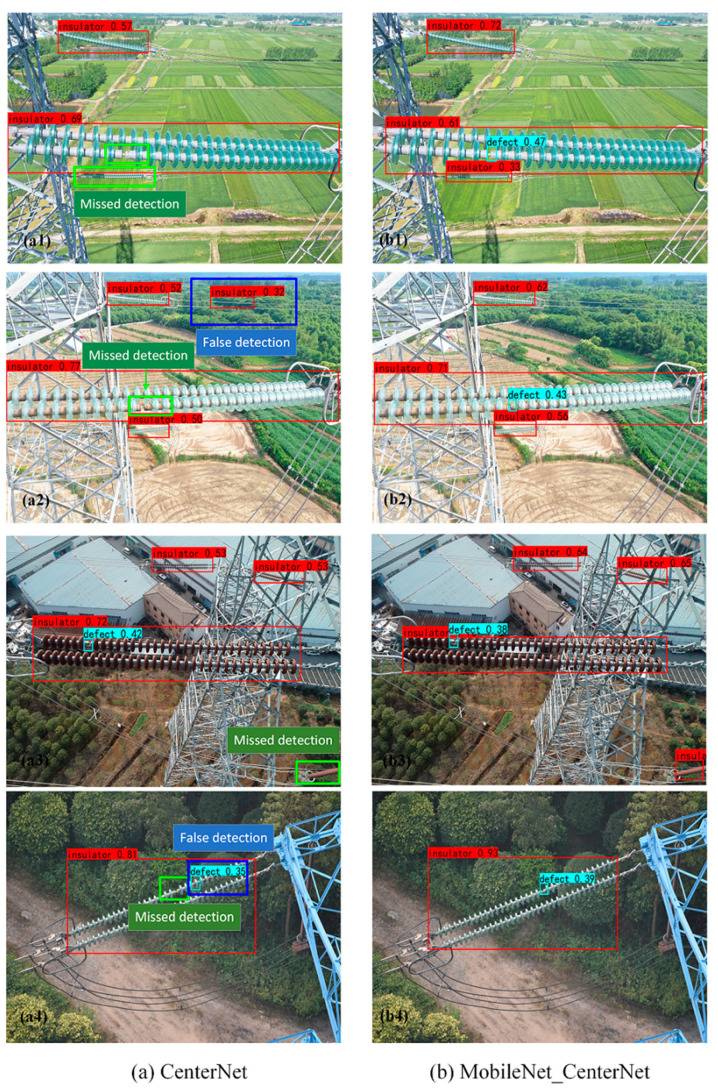
Comparison of detection results.

Figure 8 shows the test results based on the CenterNet model and MobileNet_CenterNet. The detection results based on CenterNet are shown in the first column of Figure 8. From Figure 8(a1–a4), the missed detection of insulators (blue rectangles) can be seen. In Figure 8(a2,a4) there are also cases where the insulators were incorrectly detected (green rectangles). In particular, small target insulators were missed based on CenterNet in Figure 8(a1). Figure 8(a2) shows the case of misjudging the power line as an insulator based on CenterNet. Figure 8(b1–b4) shows the detection results based on MobileNet_CenterNet. Since the model incorporates the attention mechanism module, the insulators of small targets could be accurately detected.

From the comparison results of Figure 8(a1,b1), the color of the glass insulator is similar to the background field, so it was difficult to detect the defective insulator in the picture, resulting in inaccurate positioning of the insulator by the original algorithm CenterNet. Affected by strong light and drone photography, larger insulators could be detected based on the original algorithm. However, there were still some missed detections for defect insulators of smaller sizes.

### 4.2. Comparison of Detection Effect and Computational Performance Based on Different Models

To evaluate the performance of the MobileNet_CenterNet model proposed in this study, the detection results of typical object detection models for normal insulator strings and defective insulators, including Faster-RCNN, SSD, and CenterNet, were compared using a self-built dataset, as shown in Table 4.

Among them, the software and hardware environment parameters for obtaining the inference speed FPS-CPU were: operating system Windows10, Intel(R) Core (TM) i7-10510U CPU @ 1.80 GHz, AMD Radeon (TM) RX 640, and 8 G memory.

As can be seen from Table 4, for the detection of normal insulator strings, the Precision, F1_Score, and AP based on MobileNet_CenterNet were 13.9%, 14.8%, and 21.4% higher than Faster-RCNN; 18.3%, 19.8%, and 23.3% higher than SSD; and 11.7%, 11.1%, and 21% higher than CenterNet.

For the detection of defective insulators, the Precision, F1_Score, and AP based on MobileNet_CenterNet were 13.8%, 12%, and 17.9% higher than Faster-RCNN; 18.1%, 16%, and 19.5% higher than SSD; and 11.6%, 9%, and 17.6% higher than CenterNet.

In addition, the mAP based on MobileNet_CenterNet was 17.2%, 21.5%, and 12.2% higher than Faster-RCNN, SSD, and CenterNet. Therefore, the experimental results showed that the method proposed in this study could improve the detection accuracy of the insulator state.

As could be seen from Table 4, in terms of model size, the MobileNet_CenterNet model was only 87.7 M, which is 29.6% less than CenterNet, 4.1% less than SSD, and 18.9% less than Faster-RCNN. In terms of parameters, the MobileNet_CenterNet model was 50.9% less than the original CenterNet, 33.2% less than that of SSD, and 88.3% less than that of Faster-RCNN. In particular, MobileNet_CenterNet had high detection accuracy while reducing the number of parameters, with an average precision of more than 85%.

**Figure 9:**
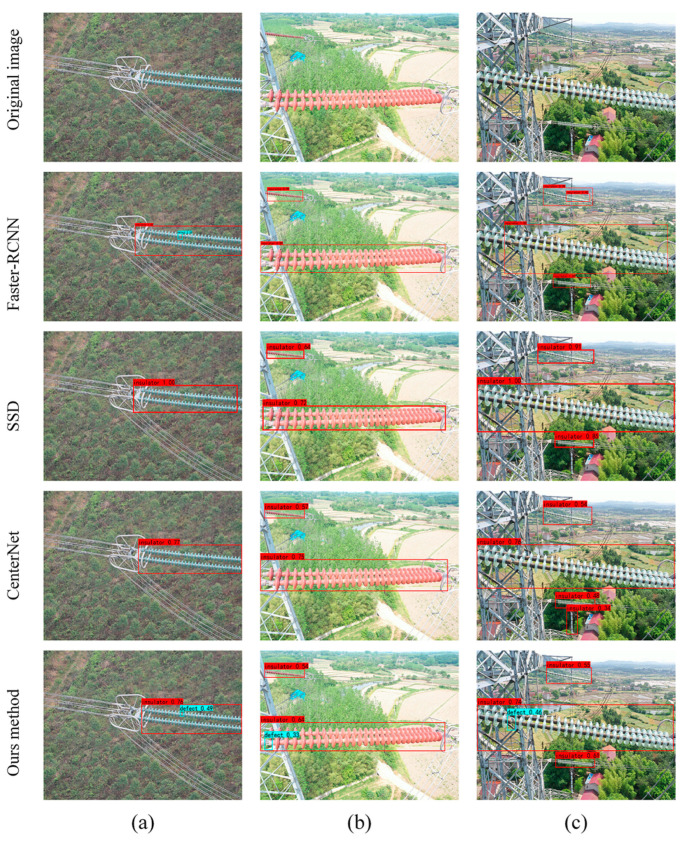
Comparison of detection results for different numbers of insulators based on different models: (**a**) small targets; (**b**) coexistence of large and small targets; and (**c**) multi-target insulators. Blue circles indicate missing insulators.

Figure 9 showed the results of testing different numbers of insulators using different models. Among them, whether it was a single insulator string or multiple insulator strings, the models based on Faster-RCNN, SSD, and CenterNet failed to accurately detect defective insulators. In addition, false detections also occurred based on Faster-RCNN and CenterNet. In addition, the models proposed in this study could accurately detect the state of the insulator.

**Figure 10:**
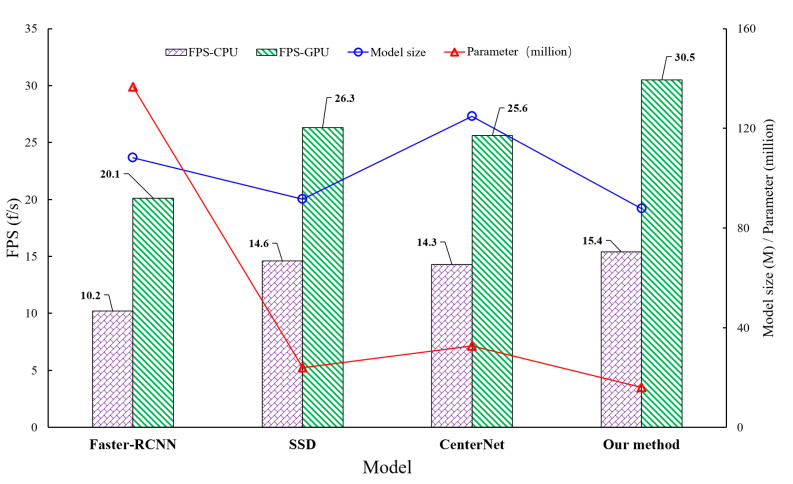
Parameter comparison of different models.

Figure 10 visually showed the different evaluation metrics of the model. In terms of inference speed, our proposed model detection was not the fastest either in the case of CPU or GPU. FPS-CPU was 10.2 f/s for Faster-RCNN, 14.6 f/s for SDD, 14.3 f/s for CenterNet, and 16.05 f/s for MobileNet_CenterNet. FPS-GPU was 20.1 f/s for Faster-RCNN, 26.3 f/s for SDD, 25.6 f/s for CenterNet, and 30.5 f/s for MobileNet_CenterNet.

Although the inference speed of the MobileNet_CenterNet model was not the best, considering the detection accuracy, size, and inference speed of the model, MobileNet_CenterNet was more suitable for the task of aerial photography insulator detection and defect detection. Therefore, the lightweight MobileNet_CenterNet model provided convenience for real-time detection of mobile devices.

### 4.3. Visualization of Insulator Feature Activation

**Figure 11:**
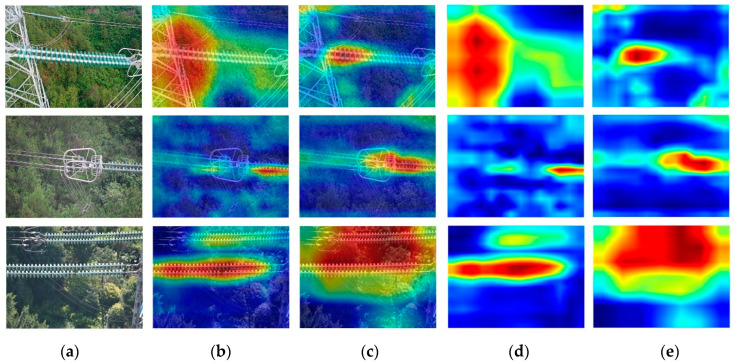
Comparison of feature activation visualizations for different models. (**a**) Original image; (**b**) CenterNet heat map; (**c**) MobileNet CenterNet heat map; (**d**) CenterNet activation map; (**e**) MobileNet CenterNet activation map.

To better reflect the performance of the optimized model, GRAD-CAM [22,38] was used to visualize the feature regions of interest in different networks, and the importance of different spatial locations was measured by gradients, including CenterNet and MobileNet_CenterNet. The feature visualization results of the two networks are compared, as shown in Figure 11. Figure 11 shows the class activation heatmap of output features of different models. The larger the range of the red area, the more features extracted by the network can cover the missing insulators that need to be identified.

Figure 11b,d shows that only a few features of the CenterNet network cover the insulator contour. Figure 11c,e shows that MobileNet_CenterNet could enable the network to have a larger and more flexible receptive field, indicating that the introduction of an attention mechanism into the network could enhance the degree of attention to missing insulators and improve the detection performance of the network.

## 5. Conclusions

To make UAVs detect insulator defects in real time and efficiently in the process of power inspection, an improved CenterNet detection model for insulator defects was proposed. In terms of feature extraction, MobileNet V1 with smaller parameters and stronger feature extraction ability was used to replace Resnet-18 in the original model, and a dual-channel attention module was introduced in the detection part so that the model could predict the target category information while considering its location information. In addition, for the problem of single-target multiple boxes caused by inaccurate center point prediction, DIoU-NMS was used to filter redundant boxes. The experimental results showed that the MobileNet_CenterNet model proposed in this paper could detect insulators with a mAP of 90.8%, FPS of 30.5 f/s, and a model size of 87.8 MB, which could detect insulator defects in aerial photography.

## Figures and Tables

**Table 1:** Dataset of CPLID + ID.

Dataset	Training Set			Validation Set			Test Set		
	Total	Default	Normal	Total	Default	Normal	Total	Default	Normal
CPLID	742	285	457	106	31	75	203	55	148
ID	543	158	385	77	37	40	160	43	117
CPLID + ID	1285	443	842	183	68	115	363	98	265

**Table 2:** Parameters of MobileNet_CenterNet model.

Module	Kernel/Stride	Input Size	Output Size
Conv_BN	(3 × 3)/2	512 × 512 × 3	256 × 256 × 32
Conv_dw	(3 × 3)/1	256 × 256 × 32	256 × 256 × 64
Conv_dw	(3 × 3)/2	256 × 256 × 64	128 × 128 × 128
Conv_dw	(3 × 3)/1	128 × 128 × 128	128 × 128 × 128
Conv_dw	(3 × 3)/2	128 × 128 × 128	64 × 64 × 256
Conv_dw	(3 × 3)/1	64 × 64 × 256	64 × 64 × 256
CBAM	(3 × 3)/1	64 × 64 × 256	64 × 64 × 256
Conv_dw	(3 × 3)/2	64 × 64 × 256	32 × 32 × 512
Conv_dw	(3 × 3)/1	32 × 32 × 512	32 × 32 × 512
Conv_dw	(3 × 3)/1	32 × 32 × 512	32 × 32 × 512
Conv_dw	(3 × 3)/1	32 × 32 × 512	32 × 32 × 512
Conv_dw	(3 × 3)/1	32 × 32 × 512	32 × 32 × 512
Conv_dw	(3 × 3)/1	32 × 32 × 512	32 × 32 × 512
CBAM	(3 × 3)/1	32 × 32 × 512	32 × 32 × 512
Conv_dw	(3 × 3)/2	32 × 32 × 512	16 × 16 × 2048
Conv_dw	(3 × 3)/1	16 × 16 × 2048	16 × 16 × 2048
CBAM	(3 × 3)/1	16 × 16 × 2048	16 × 16 × 2048
ConvTrans1	(3 × 3)/2	16 × 16 × 2048	32 × 32 × 256
ConvTrans2	(3 × 3)/2	32 × 32 × 256	64 × 64 × 128
ConvTrans3	(3 × 3)/2	64 × 64 × 128	128 × 128 × 64
Conv	(3 × 3)/1	128 × 128 × 64	128 × 128 × 64
3 × Conv	(3 × 3)/1	128 × 128 × 64	128 × 128 × 2

**Table 3:** Detection results of different datasets.

Dataset	Target	AP	Precision
CPLID	Defect	0.794	0.958
	Insulator	0.966	0.971
ID	Defect	0.817	0.973
	Insulator	0.972	0.976
CPLID + ID	Defect	0.837	0.991
	Insulator	0.979	0.989

**Table 4:** Comparison of Detection Results Based on Different Models.

Model	Evaluation Index	The Status		mAP	Model Size (MB)	Parameter (Million)	FPS-CPU (f/s)	FPS-GPU (f/s)
		**Defect**	**Insulator**					
Faster-RCNN	Precision	0.853	0.911	0.752	108.2	136.7	10.2	20.1
	F1_Score	0.69	0.852					
	AP	0.658	0.847					
SSD	Precision	0.81	0.907	0.713	91.6	24.01	14.6	26.3
	F1_Score	0.65	0.775					
	AP	0.642	0.785					
CenterNet	Precision	0.875	0.924	0.797	124.8	32.66	14.3	25.6
	F1_Score	0.72	0.902					
	AP	0.661	0.934					
Our method	Precision	0.991	0.989	0.908	87.8	16.05	15.4	30.5
	F1_Score	0.81	0.94					
	AP	0.837	0.979					

## Data Availability

The data in this paper are undisclosed due to the confidentiality requirements of the data supplier.
